# Sequence analysis and transcript expression of the *MEN1* gene in sporadic pituitary tumours

**DOI:** 10.1038/sj.bjc.6690319

**Published:** 1999-04

**Authors:** W E Farrell, D J Simpson, J Bicknell, J L Magnay, E Kyrodimou, R V Thakker, R N Clayton

**Affiliations:** Centre for Cell and Molecular Medicine, University of Keele, North Staffs Hospital, Stoke on Trent ST4 7QB, UK; MRC Molecular Endocrinology Group, MRC Clinical Sciences Centre, Imperial College School of Medicine, Hammersmith Hospital, Du Cane Road, London W12 0NN, UK

**Keywords:** *MEN1*, loss of heterozygosity, pituitary tumours, menin transcript

## Abstract

The majority of pituitary tumours are monoclonal in origin and arise sporadically or occasionally as part of multiple endocrine neoplasia type 1 (MEN1). Whilst a multi-step aetiology involving both oncogenes and tumour suppressor genes has been proposed for their development, the target(s) of these changes are less clearly defined. Both familial and sporadic pituitary tumours have been shown to harbour allelic deletion on 11q13, which is the location of the recently cloned *MEN1* gene. We investigated 23 sporadic pituitary tumours previously shown to harbour allelic deletion on 11q13 with the marker *PYGM* centromeric and within 50 kb of the *MEN1* locus. In addition, the use of intragenic polymorphisms in exon 9 and at *D11S4946*, and of telomeric loci at *D11S4940* and *D11S4936*, revealed that five of 20 tumours had loss of heterozygosity (LOH) telomeric to the menin gene. However, the overall pattern of loss in informative cases was indicative of non-contiguous deletion that brackets the menin gene. Sequence analysis of all *MEN1* coding exons and flanking intronic sequence, in tumours and matched patient leucocyte DNA, did not reveal mutation(s) in any of the 23 tumours studied. A benign polymorphism in exon 9 was encountered at the expected frequency, and in seven patients heterozygous for the polymorphism the tumour showed retention of both copies of the menin gene. Reverse transcription polymerase chain reaction analysis of ten evaluable tumours and four normal pituitaries revealed the presence of the menin transcript. Whilst these findings suggest that gene silencing is unlikely to be mechanistic in sporadic pituitary tumorigenesis, they do not exclude changes in the level or stability of the transcript or translation to mature protein. Our study would support and extend very recent reports of a limited role for mutations in the *MEN1* gene in sporadic pituitary tumours. Alternatively, these findings may point to an, as yet, unidentified tumour suppressor gene in this region. © 1999 Cancer Research Campaign


*British Journal of Cancer* (1999) **80**(1/2), 44–50
© 1999 Cancer Research Campaign
Article no. bjoc.1998.0319

### Sequence analysis and transcript expression of the *MEN1* gene in sporadic pituitary tumours

**WE Farrell[1], DJ Simpson[1], J Bicknell[1], JL Magnay[1], E Kyrodimou[1]*, RV Thakker[2] and RN Clayton[1]**

[1]Centre for Cell and Molecular Medicine, University of Keele, North Staffs Hospital, Stoke on Trent ST4 7QB, UK; [2]MRC Molecular Endocrinology Group, MRC Clinical Sciences Centre, Imperial College School of Medicine, Hammersmith Hospital, Du Cane Road, London W12 0NN, UK

**Summary** The majority of pituitary tumours are monoclonal in origin and arise sporadically or occasionally as part of multiple endocrine neoplasia type 1 (MEN1). Whilst a multi-step aetiology involving both oncogenes and tumour suppressor genes has been proposed for their development, the target(s) of these changes are less clearly defined. Both familial and sporadic pituitary tumours have been shown to harbour allelic deletion on 11q13, which is the location of the recently cloned *MEN1* gene. We investigated 23 sporadic pituitary tumours previously shown to harbour allelic deletion on 11q13 with the marker *PYGM* centromeric and within 50 kb of the *MEN1* locus. In addition, the use of intragenic polymorphisms in exon 9 and at *D11S4946*, and of telomeric loci at *D11S4940* and *D11S4936*, revealed that five of 20 tumours had loss of heterozygosity (LOH) telomeric to the menin gene. However, the overall pattern of loss in informative cases was indicative of non-contiguous deletion that brackets the menin gene. Sequence analysis of all *MEN1* coding exons and flanking intronic sequence, in tumours and matched patient leucocyte DNA, did not reveal mutation(s) in any of the 23 tumours studied. A benign polymorphism in exon 9 was encountered at the expected frequency, and in seven patients heterozygous for the polymorphism the tumour showed retention of both copies of the menin gene. Reverse transcription polymerase chain reaction analysis of ten evaluable tumours and four normal pituitaries revealed the presence of the menin transcript. Whilst these findings suggest that gene silencing is unlikely to be mechanistic in sporadic pituitary tumorigenesis, they do not exclude changes in the level or stability of the transcript or translation to mature protein. Our study would support and extend very recent reports of a limited role for mutations in the *MEN1* gene in sporadic pituitary tumours. Alternatively, these findings may point to an, as yet, unidentified tumour suppressor gene in this region.

**Keywords:** *MEN1*; loss of heterozygosity; pituitary tumours; menin transcript

Pituitary tumours are predominantly monoclonal in origin and account for approximately 10% of all intracranial neoplasms (Alexander et al, 1990; Herman et al, 1990). Although a multi-step aetiology involving both oncogenes and tumour suppressor genes (TSGs) has been proposed for the development of endocrine tumours (Melmed, 1994; Thakker, 1993), analogous to other tumour types, many of the changes are less well-defined owing, in part, to the rarity of the invasive and malignant phenotype. Thus, with the exception of the *gsp* oncogene, a constitutively active form of the $G_s$ protein occurring in up to 40% of somatotrophinomas (Landis et al, 1989; Clementi, 1990; Lyons et al, 1990; Boggild et al, 1994), only a few reports have detailed isolated instances of activating mutations in known oncogenes (reviewed by Pei and Melmed, 1996; Shimon and Melmed, 1997). Studies of known TSGs have described the loss of CDKN2/p16 expression, principally through CpG island methylation, in the majority of pituitary tumour subtypes studied (Woloschak et al, 1996, 1997; Farrell et al, 1997*a*; Simpson et al, 1999). However, loss of function of other common TSGs, as evidenced by deletion or mutation of the retinoblastoma (*Rb*) (Cryns et al, 1993; Zhu et al, 1994) or *p53* gene (Sumi et al, 1993) are infrequent events.

Deletion mapping at the site of presumed or putative TSGs has defined losses associated with particular chromosomal loci in pituitary tumours. Whilst some of these losses (chromosome 9p) appear to occur early in pituitary tumorigenesis (Farrell, 1997*b*) others are associated with the invasive and malignant phenotype including losses on chromosomes 11q13, 13q12–14, 10q26 and 1p (Bystrom et al, 1990; Boggild et al, 1994; Pei et al, 1995; Bates et al, 1997). The multiple endocrine neoplasia type 1 (*MEN1*) gene on 11q13 has now been independently cloned by two groups (Chandrasekharappa, 1997; European Consortium on MEN1, 1997*a*) and frequent germline mutations, which collectively encompass all coding exons, in familial (Agarwal et al, 1997; Chandrasekharappa et al, 1997; European Consortium on MEN1, 1997*a*; Basset et al, 1998) and sporadic forms of the disease (Agarwal et al, 1997) have been described. In addition to familial tumours, recent studies have described presumed inactivating somatic mutations in several different sporadic tumour types, including parathyroid tumours (Heppner et al, 1997), gastrinomas and insulinomas (Zhuang et al, 1997*a*) and in two of four pituitary tumours (Zhuang et al, 1997*b*) with evidence of loss of heterozygosity (LOH) within the critically deleted region on 11q13. Since we and others (Bystrom et al, 1990; Thakker et al, 1993; Boggild et al, 1994; Bates et al, 1997) have shown that a significant proportion of sporadic pituitary tumours harbour deletions (8–40%; reviewed by Thakker, 1996) that map to the critically deleted region of the *MEN1* gene, we tested the hypothesis that inactivating mutations would be found on the retained allele in these tumours. We therefore carried out complete sequence analysis of

*Received 16 June 1998*
*Revised 8 October 1998*
*Accepted 21 October 1998*

*Correspondence to:* WE Farrell

*Present address: Department of Pathology, General Hospital ‘G Gennimatas’, Athens, Greece.

**Table 1** Patient numbers, pituitary tumour subtype and radiological grade. All tumours have previously been reported as showing loss of the microsatellite marker PYGM

| Patient no. | Tumour subtype | Grade | Mutation[a] |
|---|---|---|---|
| 1 | Corticotrophinoma | 4 | – |
| 3 | Somatotrophinoma | 3 | – |
| 20 | Somatotrophinoma | 2 | – |
| 48 | Somatotrophinoma | 2 | – |
| 51 | Prolactinoma | 2 | – |
| 55 | Somatotrophinoma | 3 | – |
| 99 | Somatotrophinoma | 2 | – |
| 101 | Somatotrophinoma | 3 | – |
| 103 | Somatotrophinoma | 3 | – |
| 132 | Non-functional | 2 | – |
| 133 | Somatotrophinoma | 3 | – |
| 167 | Somatotrophinoma | 2 | – |
| 171 | Somatotrophinoma | 3 | – |
| 173 | Somatotrophinoma | 2 | – |
| 196 | Somatotrophinoma | 1 | – |
| 197 | Non-functional | 3 | – |
| 214 | Somatotrophinoma | 2 | – |
| 228 | Prolactinoma | 3 | – |
| 230 | Non-functional | 3 | – |
| 231 | Somatotrophinoma | 3 | – |
| 258 | Non-functional | 3 | – |
| 259 | Non-functional | 4 | – |
| 363 | Somatotrophinoma | 3 | – |

[a](–) absence of mutation.

all coding exons of the *MEN1* gene in 23 sporadic pituitary tumours, of various subtypes, showing LOH on 11q13. In those cases where sufficient tumour material was available, we used reverse transcription polymerase chain reaction (RT-PCR) to detect expression of the menin transcript.

### MATERIALS AND METHODS

Sufficient DNA was available from 23 pituitary tumours (15 somatotrophinomas, two prolactinomas, one corticotrophinoma and five non-functioning) and matched patient blood DNA previously shown to have sustained allelic deletion at markers tightly linked to the *MEN1* locus (Thakker et al, 1993; Boggild et al, 1994; Bates et al, 1997). Tumour samples were collected retrospectively, following standard histological assessment.

Tumours were defined as invasive or non-invasive, as previously described (Bates et al, 1997), on the basis of computerized tomography and/or magnetic resonance imaging, and graded using criteria based on a modified Hardy classification. In addition, we studied 15 histologically normal post-mortem pituitaries obtained within 12 h of death. Normal pituitaries were fixed, then embedded in paraffin and confirmed normal by routine microscopy prior to DNA extraction. Tumour number, subtype and radiological grade are shown in Table 1. Prior to the studies detailed below, all tumours and matched patient blood DNA were reassessed with the microsatellite marker *PYGM* to confirm our previous findings (Boggild et al, 1994; Thakker et al, 1993; Bates et al, 1997) of LOH at this marker. Confirmation of LOH at this marker formed the basis of the initial selection criteria for inclusion in the study.

### Tumour DNA and RNA preparation

Ten 5-µm sections were taken from the paraffin-embedded tumour. A single section was subjected to haematoxylin and eosin staining allowing tumour identification and subsequent microdissection from the remaining slides. This procedure provides a microscopically homogeneous sample with minimal contamination from non-neoplastic cells. DNA was extracted by prolonged (3–5 days) Proteinase K (0.2 mg $ml^{-1}$) digestion in 50 mM Tris-HCl (pH 8.5), 1 mM EDTA and 0.5% Tween-20. Samples were heated to 99°C for 10 min and subjected to brief centrifugation. Supernatants were removed to fresh tubes and stored at 4°C. In addition, constitutive DNA was extracted from matched blood samples using commercially available reagents (Nucleon 1; Scotlab, Strathclyde, UK).

Where sufficient suitable (< 20% contamination by normal cells) solid tumour was available, RNA was extracted using a single-step method of RNA isolation as previously described (Chomczynski and Sacchi, 1987). A portion of the solid tumour was simultaneously subject to DNA extraction (see above) and the pattern of microsatellite losses, evident in the microdissected tumours, confirmed prior to RT-PCR studies. RNA integrity was verified on 0.8% denaturing agarose gels after ethidium bromide staining.

### LOH analysis

The previously described microsatellite marker *PYGM* and other markers within the 11q13 critically deleted region (Manickam et al, 1997), inferred from LOH studies, were used to determine the pattern and extent of allelic deletion. The microsatellite markers and their relative position are shown in Figure 3. The markers *PYGM* and *D11S4936* define the centromeric and telomeric extent of a 300 kb interval (respectively) that encompasses the *MEN1* gene. *D11S4946* is a *MEN1* intragenic marker and *D11S4940* lies within this interval (Figure 3). Primer sequences were obtained from the Genome DataBase. PCRs were carried out in 25-µl volumes with 1.5 mM magnesium chloride, 200 µM each of dATP, dGTP, dTTP and dCTP, 2 pmol of each primer, template DNA (as serial dilutions) and 1 unit of *Taq* DNA polymerase. PCR was carried out for 25–30 cycles. Constitutive and tumour DNA products were run adjacently and separated on 8% non-denaturing polyacrylamide gels, fixed in 10% methylated spirit/0.5% acetic acid for 6 min and then incubated in 0.1% aqueous silver nitrate for 15 min. Following two brief washes in distilled water, products were visualized by development in 1.5% sodium hydroxide/0.1% formaldehyde.

In addition, for the microsatellite markers in which one of the primers is located within an Alu repeat (*D11S4946*, *D11S4940* and *D11S4936*) (Manickam et al, 1997), LOH analysis was also assessed by $\gamma^{32}$P-ATP end-labelling of the non-Alu primer. Where assessable, both techniques of visualization were concordant for assignment of LOH status. However, considerably fewer of the cohort gave resolvable bands that were assessable by the end-labelling techniques than by silver staining, we therefore used the latter technique for all of the markers employed in this study.

Allelic loss was identified by a reduction in band intensity of greater than 80% or the absence of one of the expected PCR products in the amplified DNA. Template DNA was serially diluted prior to PCR amplification, allowing a direct comparison of dilutions that produced similar product intensities between constitutive DNA and the retained allele(s) in the matched tumour sample.

### *MEN1* sequence analysis

The *MEN1* gene comprises ten exons (exon 1 is not translated). Sequencing reactions of all coding exons encompassed

© Cancer Research Campaign 1999

exon–intron boundaries using, in total, 15 primer pairs as described by the European Consortium on MEN1 (1997*a*). PCR products were directly sequenced using a standard dideoxy protocol (USB; Sequenase Version 2.0 DNA Sequencing Kit) and normal and tumour DNA sequences were compared. All 23 tumours were subjected to sequence analysis of the nine coding exons using both sense and antisense oligonucleotide primers.

### RT-PCR analysis

Sufficient RNA was available from ten of the tumours shown to harbour allelic deletion within the 11q13 *MEN1* interval. A total of 2–5 μg of RNA was heated to 65°C for 10 min in the presence of 0.2 μg random hexamer primer and then snap-cooled on ice in a total volume of 12 μl. In a final volume of 20 μl, the reaction mixture contained 200 μM of dATP, dGTP, dTTP and dCTP, 1X first strand buffer mix, 10 mM dithiothreitol and 2 units of reverse transcriptase (Superscript II Rnase H-Reverse Transcriptase; GibcoBRL, Life Technologies Ltd, UK). The reaction mixtures were then incubated at 25°C for 10 min and 42°C for 1 h as described by the manufacturers. The reaction was stopped by heating to 95°C for 5 min and cDNAs stored at –20°C. PCR amplification of the resulting cDNA was achieved with primers designed to encompass intron 8 of the *MEN1* gene to distinguish possible contamination by genomic DNA. The primer sequences used comprised an exon 8 upstream primer, CCAATGATGT-CATCCCCAA, and exon 9 downstream primer, CCTG-GAGGGCGGAACCT. PCR amplification was limited to 30 cycles to ensure amplification in the exponential phase. In addition, identical conditions were used for both normal and tumourous pituitaries, thus allowing semi-quantitative comparison relative to an *HPRT* internal control (see below). Visualization of the resulting 115 bp product was carried out as described above. To confirm cDNA integrity, in the tumour samples, a portion of the housekeeping gene *HPRT* was co-amplified from tumour cDNA, forward primer TGACCAGTCAACAGGGGACA, reverse primer GCCTGACCAAGGAAAGCAAAGTC (product size 135 bp). In all cases appropriate controls, including omission of reverse transcriptase, failed to produce an amplified product.

### RESULTS

We first analysed 23 pituitary tumours and matched leucocyte DNA for LOH on chromosome 11q13 within the minimal interval of the *MEN1* gene inferred by LOH studies (Manickam et al, 1997). Tumour subtype and grade are shown in Table 1 and have been previously investigated by us in several recent reports (Boggild et al, 1994; Thakker et al, 1993; Bates et al, 1997; Farrell et al, 1997*b*). Tumours were initially selected on the basis of LOH at the polymorphic marker *PYGM*, located centromeric and within 50 kb of the *MEN1* gene (European Consortium on MEN1, 1997*b*). Using recently described microsatellite markers in the *MEN1* interval (Manickam et al, 1997; Figure 3) we determined which of these 23 tumours had sustained loss at the intragenic locus, *D11S4946*, and the telomeric loci, *D11S4940* and *D11S436*. Tumours were initially re-evaluated with the marker *PYGM* to confirm our previous reports of LOH at this marker (Thakker et al, 1993; Boggild et al, 1994; Bates et al, 1997). Further LOH studies of these tumours were undertaken. *D11S4936* located at the telomeric boundary of the interval showed LOH in three of

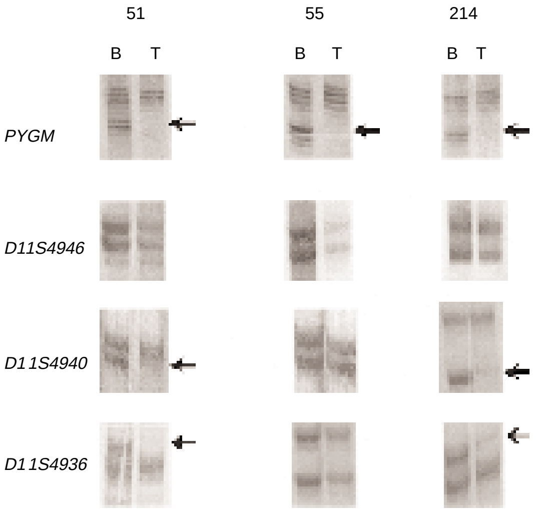

**Figure 1** Representative examples of LOH in sporadic pituitary tumours. All tumours in the study show loss of one copy of the marker *PYGM*. In addition, the Figure shows examples of LOH at other markers in the critically deleted region inferred from LOH studies. In all cases, DNA from microdissected tumours are compared to matched patient leucocyte DNA and run adjacently. Tumour numbers are shown above the lanes and the microsatellite markers on the left of each matched tumour normal DNA pair. Arrows show LOH as a substantial decrease in the intensity of one of the alleles in the tumour DNA relative to the matched leucocyte DNA. The absence of an arrow indicates retention of heterozygosity in the tumour

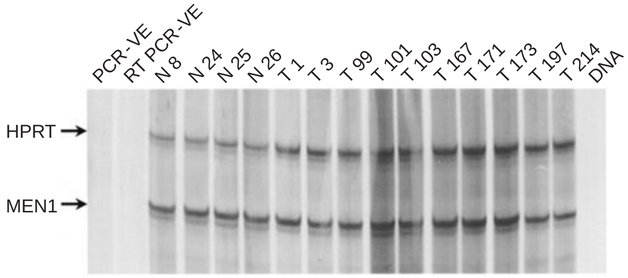

**Figure 2** Representative examples of RT-PCR analysis of a portion of the menin transcript in tumours showing LOH on 11q13 and from normal pituitaries. The position of the amplified menin product (115 bp) and the co-amplified *HPRT* transcript (135 bp) are shown on the left of the figure and are arrowed

13 informative cases, and *D11S4940*, in five of 14 informative cases (Figure 1 and summarized in Figure 3). Overall, the data show that, of the 23 tumours with evidence of LOH at *PYGM*, five tumours had sustained deletion at one or more markers that lie telomeric to *PYGM*. However, in two of these tumours (51 and 214) that were informative for the intragenic marker *D11S4946* and showed retention of heterozygosity, the overall pattern of loss demonstrates non-contiguous deletion that brackets, but excludes, the *MEN1* gene. The other two tumours, informative for *D11S4946* (55 and 231), also showed retention of both copies of the menin gene, and in these cases loss were confined to the marker *PYGM*. Since the more distal markers to *PYGM* were either frequently uninformative or did not yield an evaluable product, we attempted to use the other recently described markers in this region (Manickam et al, 1997). Despite repeated attempts under different conditions, we were unable to resolve assessable PCR products

 *© Cancer Research Campaign 1999*

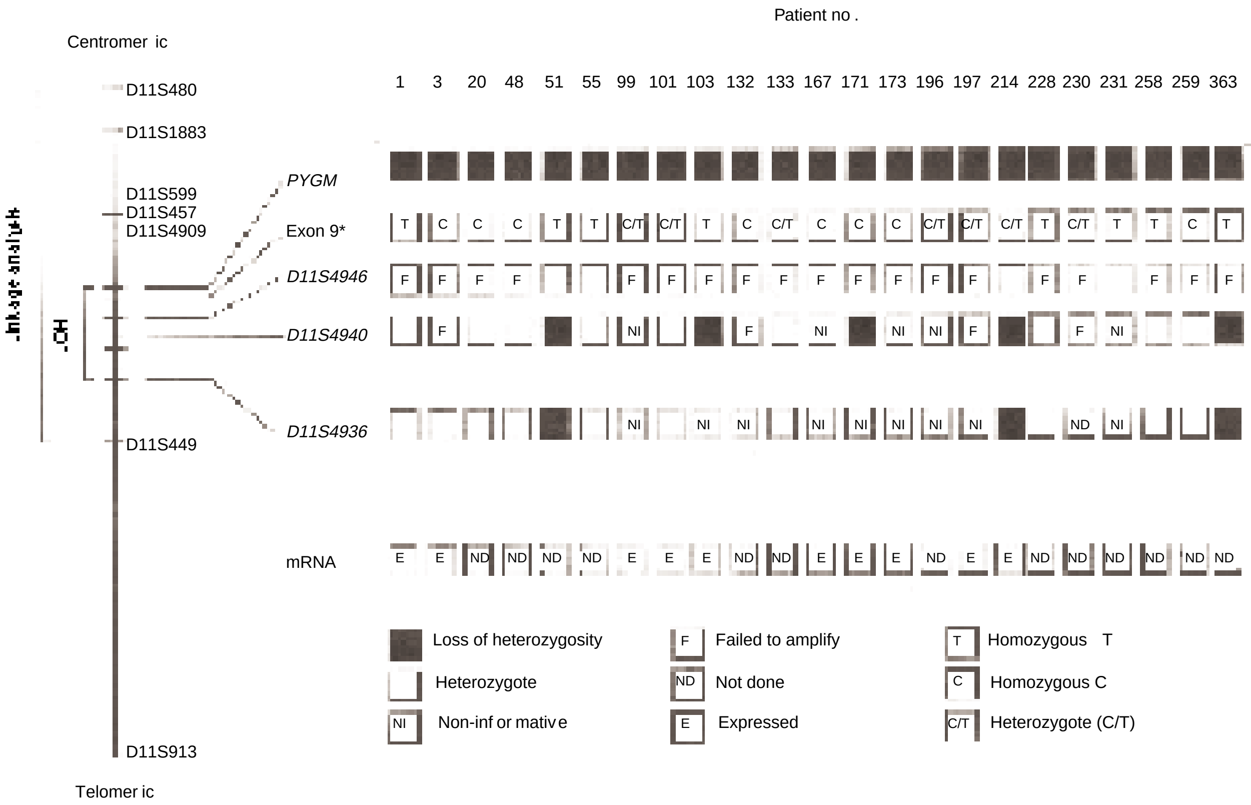

**Figure 3** Summary chart of 11q13 allelic status in 23 pituitary tumours. The left of the Figure is a schematic representation of a portion of chromosome 11q where the crossbars represent the approximate map positions of the microsatellite markers. The markers used in this study, *PYGM* to *D11S4936*, define the centromeric and telomeric (respectively) boundary of the critically deleted region inferred from LOH studies. In addition, the Figure shows the status of the exon 9 polymorphism in all tumours included in the study (*) and the RT-PCR summary for ten tumours investigated. Patient numbers are shown at the top of the chart from either tumour or leucocyte DNA (data not shown). No losses, at any of the microsatellite markers used in this study, were found in any of 15 normal pituitaries evaluated (data not shown).

The presence of somatic mutations in the gene encoding menin was investigated by direct sequence analysis of all coding exons together with their flanking intronic sequences. No mutations were detected in any of the 23 tumours studied and, in all cases, tumours were analysed and compared to matched patient leucocyte DNA. A published polymorphism in exon 9 (D418, GAC/GAT, 54% C: 42% T; Agarwal et al, 1997; Chandrasekharappa et al, 1997; Bassett et al, 1998) was encountered at the expected frequency and was identical in DNA from tumours and matched leucocytes. In five of the 23 cases, both tumour and matched leucocyte DNA (99, 101, 133, 196, 197, 214, 230) were heterozygous for the polymorphism. Thus, collectively, the presence of either the exon 9 polymorphism and/or retention of heterozygosity for the *MEN1* intragenic marker *D11S4946* demonstrated retention of both alleles of the menin gene in ten of 23 tumours studied.

Since we were unable to detect mutation(s) in the coding region of the menin gene, alternative mechanisms of gene silencing found in other tumour types, including mutation in the promoter region or hypermethylation of a CpG island, were indirectly investigated. For ten of the tumours in this study, sufficient solid tumour was available for RT-PCR analysis. Microscopically, the tumours comprise > 80% tumour cells and, prior to RT-PCR analysis, we re-assessed their LOH status. All solid tumour showed the same pattern of LOH as their microdissected counterparts, thus demonstrating minimal contamination from surrounding or infiltrating ‘normal’ cells. In all ten cases, and in four histologically normal pituitaries, we detected the presence of a portion of the menin transcript (Figure 2 and summarized in Figure 3). Whilst we did not attempt to quantify transcript expression levels, a similar signal intensity was apparent in both tumourous and normal pituitaries when the co-amplified signal for *HPRT* is taken into account. These results would suggest that the menin transcript in the tumour samples was not simply a consequence of a ‘leaky gene’.

### DISCUSSION

Previous studies by our own and other groups have described 11q13 LOH in sporadic (Bystrom et al, 1990; Boggild et al, 1994; Thakker et al, 1993; Bates et al, 1997; Zhuang et al, 1997*b*) and MEN1-associated pituitary adenomas (Dong et al, 1997). In addition to pituitary tumours other sporadic endocrine tumours not associated with MEN1 show variable, often frequent, LOH (3–78%) on 11q13 (Friedman et al, 1992; Lida et al, 1995; Jakobovitz et al, 1996; Debelenko et al, 1997). The recent cloning of the *MEN1* gene and identification of germline mutations, in both familial and sporadic forms of this disease (Agarwal et al, 1997; Chandrasekharappa et al, 1997; European Consortium on MEN1, 1997*a*; Bassett et al, 1998), has facilitated the identification of mutations in tumours not associated with the MEN1

© Cancer Research Campaign 1999 

syndrome. To this end, somatic mutations have recently been identified in sporadic tumours of the parathyroid (54%) (Heppner et al, 1997), gastrinomas and insulinomas (33% and 17% respectively) (Zhuang et al, 1997*b*) with evidence, in the majority of cases, of LOH within the critically deleted region on 11q13. Two studies of sporadic pituitary tumours have been reported to date (Zhuang et al, 1997*b*; Prezant et al, 1998). In the first report, *MEN1* mutations were found in two of 37 sporadic pituitary tumours investigated (Zhuang et al, 1997*b*); however, the second study failed to detect mutation in any of 45 tumours investigated (Prezant et al, 1998).

In this study we further investigated both clinically active and inactive pituitary tumours with evidence of LOH at the microsatellite marker *PYGM*. The combined data showed that, of 23 tumours with evidence of LOH at *PYGM*, five had sustained concomitant loss at one or more distal (telomeric) markers within the 300 kb interval. However, in those cases informative for the intragenic marker *D11S4946* and/or the exon 9, polymorphism retention of heterozygosity defines either a region of non-contiguous deletion or the deletions telomeric extent. Non-contiguous deletions are not without precedent in sporadic pituitary tumours since in a previous report of LOH on chromosome 9p we identified non-contiguous deletions that bracket, but specifically exclude, the TSGs p15 and p16 (Farrell et al, 1997*b*).

Complete sequence analysis of all coding exons, of the gene responsible for MEN1, with evidence of LOH within this region, did not reveal mutation(s) in any of the 23 tumours studied. In other studies of sporadic tumours reported to date (Heppner et al, 1997; Zhuang et al, 1997*a*, 1997*b*; Bassett et al, 1998; Prezant et al, 1998) sequence abnormalities were initially detected either by dideoxy fingerprinting (Heppner et al, 1997; Prezant et al, 1998) or SSCP analysis (Zhuang et al, 1997*a*, 1997*b*; Basset et al, 1998) respectively. Although both of these techniques facilitate rapid screening both may fail to detect mutations. Our direct sequencing strategy circumvented the inherent problems associated with these two techniques and it is therefore unlikely that we had failed to identify mutations present within the coding exons or their flanking intronic sequences. The reliability of our sequencing data was further supported by the detection of a benign polymorphism in exon 9 of the *MEN1* gene, showing complete concordance between tumours and matched leucocyte DNA. In seven cases that were heterozygous for this polymorphism, this showed retention of both copies of the menin gene. The combined data of tumours heterozygous for the polymorphism and/or retention of the intragenic marker (*D11S4946*) show that, in ten of the tumours studied, both copies of the menin gene are retained. Thus, whilst all tumours were initially selected on the basis of LOH at the microsatellite marker *PYGM* that lies within 50 kb of the *MEN1* locus, in these cases it was not accompanied by loss of either copy of the menin gene. The cloning and identification of the gene responsible for MEN1 has been especially challenging, due in part to this region being particularly gene-rich. Our results may point to the involvement of another TSG in this region in pituitary tumours and perhaps in other sporadic endocrine tumour types with evidence of LOH on 11q13. The recently described microsatellite markers in the *MEN1* region and other markers on this chromosomal arm (European Consortium on MEN1, 1997*b*; Manickam et al, 1997) will substantially aid the identification of potentially novel genes and their protein products with tumour suppressor activity in sporadic tumours.

The first report describing mutations of the *MEN1* gene in sporadic pituitary tumours (Zhuang et al, 1997*b*) initially screened 38 tumours for allelic deletion using either interphase fluorescence in situ hybridization (FISH) or microsatellite analysis. Collectively, the two techniques identified deletions in four tumours (two by FISH and two by LOH analysis). However, only the tumours showing LOH by microsatellite analysis were found to harbour mutations. The discordance between this first report and the present study is difficult to explain since we specifically targeted only those tumours with evidence of LOH (as defined by microsatellite analysis) within the critically deleted region of the *MEN1* interval, for sequence analysis. The difference may be partly explained by the number of tumours within each particular subtype. The majority of tumours in our study comprised somatotrophinomas, since these show the highest frequency of LOH on 11q13. However, in the study by Zhuang et al (1997*b*), significantly more corticotrophinomas were studied, of which, the one shown to harbour allelic deletion had also sustained a mutation in the retained allele. Our study would discount a role for mutation in the menin gene in pituitary tumours with evidence of LOH on 11q13 and in somatotrophinomas in particular. Indeed, the only other report of sporadic pituitary tumours (Prezant et al, 1998) also failed to detect mutation in the menin gene in 45 tumours investigated. In the other two studies of sporadic tumours reported to date, deletions were assigned either by microsatellite analysis (parathyroid tumours; Heppner et al, 1997) or interphase FISH (gastrinomas and insulinomas; Zhuang et al, 1997*a*). Whilst both techniques identified tumours that were then found to have sustained a mutation in the remaining allele, they also identified a significant number of tumours with allelic loss that was not accompanied by mutation in the remaining allele.

In the study reported by Zhuang et al (1997*b*), two sporadic pituitary tumours had loss of one copy of the *MEN1* gene without gene mutation in the remaining copy. In both cases, loss was assigned using interphase FISH whilst in the two tumours shown to have sustained mutation allelic deletion was determined by microsatellite analysis. Among the possible reasons these authors propose for the absence of mutations in these tumours is that they may contain mutations in promoter regions of the *MEN1* gene that was not screened or that alternative mechanisms, such as hypermethylation of a CpG island, may inhibit transcription of the *MEN1* gene in these tumours. To determine the role of alternate mechanisms of gene silencing we used RT-PCR analysis. In both normal and tumourous pituitaries the presence of the menin transcript was readily detectable and similar results are reported by Prezant et al (1998). The role of methylation as a mechanism of gene silencing in pituitary tumours has very recently been established (Farrell et al, 1997*a*; Woloschak et al, 1997; Simpson et al, 1999). These two studies have shown frequent inactivation of the p16 gene by CpG island methylation, whilst in an earlier study we showed frequent non-contiguous deletion on chromosome 9p in pituitary tumours that bracketed, but excluded, the p16 gene (Farrell et al, 1997*b*). Whilst our study did not reliably allow us to draw any conclusions regarding the relative levels of transcription between normal and tumourous tissue, they support the view that mutation in non-coding *MEN1* sequences or epigenetic mechanisms, such as hypermethylation are unlikely to play a role in transcriptional silencing. However, the availability of antibodies to the menin protein may well define reduced expression of this protein or loss through aberrations in post-transcriptional processing of the menin transcript as mechanistic in pituitary tumorigenesis.

© Cancer Research Campaign 1999

In summary, our study shows an absence of mutation in the coding region of the *MEN1* gene in sporadic pituitary tumours of various subtypes despite loss of microsatellite markers within the critically deleted region encompassing this gene. The menin transcript was readily detectable in the tumours examined suggesting that gene silencing is unlikely to be mechanistic in pituitary tumorigenesis.

### ACKNOWLEDGEMENTS

We wish to thank the Association for International Cancer Research (AICR) for financial support. DJS is the recipient of a West Midlands New Blood Training Fellowship and their support is gratefully acknowledged.

### REFERENCES

Agarwal SK, Kester MB, Debelenko LV, Heppner C, Emmert-Buck MR, Skarulis MC, Doppman JL, Kim YS, Lubensky IA, Zhengping Z, Green JS, Guru SC, Manickam P, Olufemi S-E, Liotta LA, Chandrasekarappa SC, Collins FS, Spiegal AM, Lee Burns A and Marx SJ (1997) Germline mutations of the *MEN1* gene in familial multiple endocrine neoplasia type 1 and related states. *Hum Mol Genet* **6**: 1169–1175

Alexander JM, Biller BMK, Bikkal H, Zervas NT, Arnold A and Klibanski A (1990) Clinically non-functioning pituitary tumors are monoclonal in origin. *J Clin Invest* **86**: 336–340

Bassett JHD, Forbes SA, Pannett AAJ, Lloyd SE, Christie PT, Wooding C, Harding B, Besser GM, Monson JP, Sampson J, Wass AH, Wheeler MH and Thakker RV (1998) Characterization of mutations in patients with multiple endocrine neoplasia type 1. *Am J Hum Genet* **62**: 232–244

Bates AS, Farrell WE, Bicknell EJ, Talbot AJ, Broome JC, Perrett CW, Thakker RV and Clayton RN (1997) Allelic deletion in pituitary adenomas reflects aggressive biological activity and has potential value as a prognostic marker. *J Clin Endocrinol Metab* **82**: 818–824

Boggild MD, Jenkinson S, Pistorello M, Boscaro M, Scanarini M, McTernan, Perret CW, Thakker RV and Clayton RN (1994) Molecular genetic studies of sporadic pituitary tumours. *J Clin Endocrinol Metab* **78**: 387–392

Bystrom C, Larsson C, Blomberg C, Sandelin K, Falkmer E, Skogseid B, Oberg K, Werner S and Nordenskjold M (1990) Localisation of the *MEN1* gene to a small region within chromosome 11q13 by deletion mapping in tumours. *Proc Natl Acad Sci USA* **87**: 1968–1972

Chandrasekharappa SC, Guru SC, Manickam P, Olufemi S-E, Collins FS, Emmert-Buck MR, Debelenko LV, Zhuang Z, Lubensky IA, Liotta LA, Crabtree JS, Wang Y, Roe BA, Weisemann J, Boguski MS, Agerwal SK, Kester MB, Kim YS, Heppner C, Dong Q, Spiegal Am, Burns AL and Marx SJ (1997) Positional cloning of the gene for multiple endocrine neoplasia-type 1. *Science* **276**: 404–407

Chomczynski P and Sacchi N (1987) Single step method of RNA isolation by acid guanidine thiocyanate extraction. *Anal Biochem* **162**: 156–159

Clementi E, Malgaretti N, Meldolesi J and Taramelli R (1990) A new constitutively activating mutation in $G_s$ protein α subunit-*gsp* oncogene is found in human pituitary tumours. *Oncogene* **5**: 1059–1061

Cryns V, Alexander JM, Klibanski A and Arnold A (1993) The retinoblastoma gene in human pituitary tumors. *J Clin Endocrinol Metab* **77**: 644–646

Debelenko LV, Zhuang Z, Emmert-Buck MR, Chandrasekharappa SC, Manickam P, Guru SC, Marx Sj, Skarulis MC, Spiegal AM, Collins FS, Jenson RT, Liotta LA and Lubensky IA (1997) Allelic deletion on chromosome 11q13 in MEN1-associated and sporadic gastrinomas and pancreatic endocrine tumours. *Cancer Res* **57**: 2238–2243

Dong Q, Debelenko LV, Chadrasekharappa SC, Emmert-Buck MR, Zhuang Z, Guru SC, Manickam P, Skarulis M, Lubensky IA, Liotta LA, Collins FS, Marx SJ and Spiegal AM (1997) Loss of heterozygosity at 11q13: analysis of pituitary tumours, lung carcinoids, lipomas and other uncommon tumours in subjects with familial multiple endocrine neoplasia type 1. *J Clin Endocrinol Metab* **82**: 1416–1420

European Consortium on MEN1 (1997*a*) Identification of the multiple endocrine neoplasia type 1 (*MEN1*) gene. *Hum Mol Genet* **6**: 1177–1183

European Consortium on MEN1 (1997*b*) Construction of a 1.2Mb sequence ready contig of chromosome 11q13 encompassing the multiple endocrine neoplasia type 1 (*MEN1*) gene. *Genomics* **44**: 94–100

Farrell WE, Simpson DJ, Bicknell J, McNicol AM, Clayton RN (1997*a*) Methylation of the tumour supressor gene (TSG) CDKN2/MTS1 occurs frequently in invasive and non-invasive pituitary adenomas and is associated with loss of p16 expression. Mutation Detection 4th International Workshop; Human Genome Organisation, Brno Czech Republic. (abstract): 25

Farrell WE, Simpson D, Bates AS, Talbot JA, Bicknell J and Clayton RN (1997*b*) Chromosome 9 p deletions in invasive and non invasive non-functional pitutary adenomas: The deleted region involves markers outside of the MTS1 and MTS2 gene. *Cancer Res* **57**: 2703–2709

Friedman E, DE Marco L, Gejman PV, Norton JA, Bale AE, Aurbach GD, Spiegal AM and Marx SJ (1992) Allelic loss from chromosome 11 in parathyroid tumours. *Cancer Res* **52**: 6804–6809

Heppner C, Kester MB, Agarwal SK, Debelenko LV, EmmertBuck MR, Guru SC, Manickam P, Olufemi SE, Skarulis MC, Doppman JL, Alender RH, Kim YS, Saggar SK, Lubensky IA, Zhuang ZP, Liotta LA, Chandrasekharappa SC, Collins FS, Spiegel AM, Burns AL and Marx SJ (1997) Somatic mutations of the *MEN1* gene in parathyroid tumours. *Nature Gen* **16**: 375–378

Herman V, Fagin J, Gonsky R, Kovaks K and Melmed S (1990) Clonal origin of pituitary adenomas. *J Clin Endocrinol Metab* **71**: 1427–1433

Jakobovitz O, Nass D, DeMarco L, Barbosa AJA, Simoni FB, Rechavi G and Friedman (1996) Carcinoid tumours frequently display genetic abnormalities involving chromosome 11. *J Clin Endocrinol Metab* **81**: 3164–3167

Landis CA, Masters SB, Spada A, Pace AM, Bourne HR and Vallar L (1989) GTPase inhibiting mutations activate the alpha chain of $G_s$ and stimulate adenyl cyclase in human pituitary tumours. *Nature* **340**: 692–696

Lida A, Blake K, Tunny T, Klemm S, Stowasser M, Hayward N, Gordon R, Nakamura Y and Imai T (1995) Allelic losses on chromosome band 11q13 in aldosterone-producing adrenal tumours. *Genes Chromosomes Cancer* **12**: 73–75

Lyons J, Landis CA, Harsh G, Harsh G, Vallar L, Grunewald K, Feichtinger H, Duh QY, Clarke OH, Kawasaki E, Bourne HR and McCormick (1990) Two G protein oncogenes in human endocrine tumours. *Science* **249**: 655–659

Manickam P, Guru SC, Debelenko LV, Agarwal SK, Olufemi S-E, Weisemann JM, Boguski MS, Crabtree JS, Wang YP, Roe BA, Lubensky IA, Zhuang ZP, Kester MB, Burns AL, Spiegel AM, Marx SJ, Liotta LA, EmmertBuck MR, Collins FS and Chandrasekharappa SC (1997) Eighteen new polymorphic markers in the multiple endocrine neoplasia type 1 (*MEN1*) region. *Hum Genet* **101**: 102–108

Melmed S (1994) Pituitary neoplasia. In: *Multiple Endocrine Neoplasia*, Gagel RF (ed). *Endocrinol Metab Clinics North Am* **23**: 81–92

Pei L, Melmed S, Scheithauer B, Kovaks K, Benedict WE and Prager D (1995) Frequent loss of heterozygosity at the retinoblastoma susceptibility gene (RB) locus in aggressive pituitary tumors: evidence for a chromosome 13 tumour supressor gene other than RB. *Cancer Res* **55**: 644–646

Pei L and Melmed S (1996) Oncogenes and tumour suppressor genes in pituitary tumorigenesis. In *Oncogenesis and Molecular Biology of Pituitary Tumours*, Melmed S (ed) *Front Horm Res* **20**: 122–136

Prezant TR, Levine J and Melmed S (1998) Molecular characterization of the *MEN1* tumour suppressor gene in sporadic pituitary tumors. *J Clin Endocrinol Metab* (in press)

Simpson DJ, Bicknell JE, McNicol AM, Clayton RN, Farrell WE (1999) Hypermethylation of the p16/CDKN2A/MTS1 gene and loss of protein expression is associated with non-functional pituitary adenomas but not somatotrophinomas. *Genes, Chromosomes Cancer* **24**: 328–336

Shimon I and Melmed S (1997) Genetic basis of endocrine disease: pituitary tumor pathogeneis. *J Clin Endocrinol Metab* **82**: 1675–1681

Sumi T, Stefaneanu L, Kovaks K, Asa S and Rindi G (1993) Immunohistochemical study of p53 protein in human and animal pituitary tumours. *Endocrinol Pathol* **4**: 95–99

Thakker RV (1993) The molecular genetics of the multiple endocrine neoplasia syndrome. *Clin Endocrinol* **38**: 1–14

Thakker RV (1996) Role of chromosome 11 in hereditary and sporadic pituitary tumourigenesis. In *Front Hormone Research*, Melmed S (ed). Basel, Karger. **20**: 179–193

Thakker RV, Pook MA, Wooding C, Boscaro M, Scanarini M and Clayton RN (1993) Association of somatotrophinomas with loss of alleles on chromosome 11 with gsp mutations. *J Clin Invest* **91**: 2815–2821

Woloschak M, Yu A, Xiao J and Post KD (1996) Frequent loss of the $p16^{INK4a}$ gene product in human pituitary tumours. *Cancer Res* **56**: 2493–2486

Woloschak M, Yu A and Post KD (1997) Frequent inactivation of the *p16* gene in human pituitary tumours by gene methylation. *Mol Carcinogen* **19**: 221–224

Zhu J, Leon SP, Beggs AH, Busque L, Gilliland DG and Black PM (1994) Human pituitary adenomas show no loss of herozygosity at the retinoblastoma gene locus. *J Clin Endocrinol Metab* **78**: 922–927

© Cancer Research Campaign 1999 

Zhuang ZP, Vortmeyer AO, Pack S, Huang S, Pham TA, Wang CY, Park WS, Agarwal SK, Debelenko LV, Kester M, Guru SC, Manickam P, Olufemi SE, Yu F, Heppner C, Crabtree JS, Skarulis MC, Venzon DJ, EmmertBuck MR, Spiegel AM, Chandrasekhrapper SC, Collins FS, Burns AL, Marx SJ, Jensen RT, Liotta LA and Lubensky (1997*a*) Somatic mutations of the *MEN1* tumour suppressor gene in sporadic gastrinomas and insulinomas. *Cancer Res* **57**: 4682–4686

Zhuang Z, Ezzat SZ, Vortmeyer AO, Weil R, Oldfield EH, Park SP, Pack S, Huang S, Agarwal SK, Guru SC, Manickam P, Debelenko LV, Kester MB, Olufemi SE, Heppner C, Crabtree JS, Burns AL, Spiegel AM, Marx SJ, Chandrasekharappa SC, Collins FS, Emmert-Buck MR, Liotta LA, Asa SL and Lubensky IA, Walker LM, Holm A, Oberg A, Sundqvist T, El Haj AJ (1997*b*) Mutation of the *MEN1* tumour suppressor gene in pituitary tumours. *Cancer Res* **57**: 5446–5451

 *© Cancer Research Campaign 1999*
